# Supplementary material for: Economically viable geological CO2 storage from direct air capture has critical threshold of 70% CO2 concentration
Source: Commun Eng. 2025 Jul 15;4:127. doi: 10.1038/s44172-025-00468-5 (PMC12264126; doi:10.1038/s44172-025-00468-5)
Supplement: Supplementary file 2 — Supplementary Information [file 44172_2025_468_MOESM2_ESM.pdf]

Supplementary Information

Economically Viable Geological CO_2 Storage from Direct Air Capture has Critical Threshold of 70% CO_2 Concentration

Le Zhang, Yunfeng Liang, Arata Kioka and Takeshi Tsuji**

School of Engineering, The University of Tokyo, Tokyo 113-8656, Japan

*Corresponding author

E-mail: liang@sys.t.u-tokyo.ac.jp (Y. Liang)

E-mail: tsuji@sys.t.u-tokyo.ac.jp (T. Tsuji)

Supplementary Note 1: Radial Distribution Function (RDF) and Coordination Number Analysis

Besides the Lennard-Jones (LJ) potential and Coulomb potential investigation, we further analyzed the radial distribution functions (RDF) and coordination numbers (CN) for CO_2-CO_2, N_2-N_2, and CO_2-N_2 pairs within the system. Supplementary Figure 1 a), d), and g) present the RDF distributions for CO_2-CO_2, N_2-N_2, and CO_2-N_2, respectively. The RDF peak values for different CO_2 concentrations were plotted against temperature, resulting in the curves shown in Supplementary Figure 1 b), e), and h). The CN values corresponding to the RDF valley following the first peak were also calculated and are presented in Supplementary Figure 1 c), f), and i). The peak data are influenced by concentration, leading to instability. For instance, Supplementary Figure 1 e) shows significant fluctuations in the RDF peak of N_2-N_2 under 90% CO_2 conditions, while the CO_2-CO_2 data remain stable. The CN trends displayed in Supplementary Figure 1 c), f), and i) reveal that CO_2-CO_2 has a larger slope compared to N_2-N_2 and CO_2-N_2, which show relatively minor changes in slope. A similar trend is observed in the first peak height plots, where the slope changes for N_2-N_2 and CO_2-N_2 are less pronounced. Notably, in the first peak height distribution of CO_2-CO_2, we observed a slope change similar to that in. The difference in values between the right and left sides of the plot is approximately 40.2%, consistent with the previously observed trends in density distribution and LJ potential.

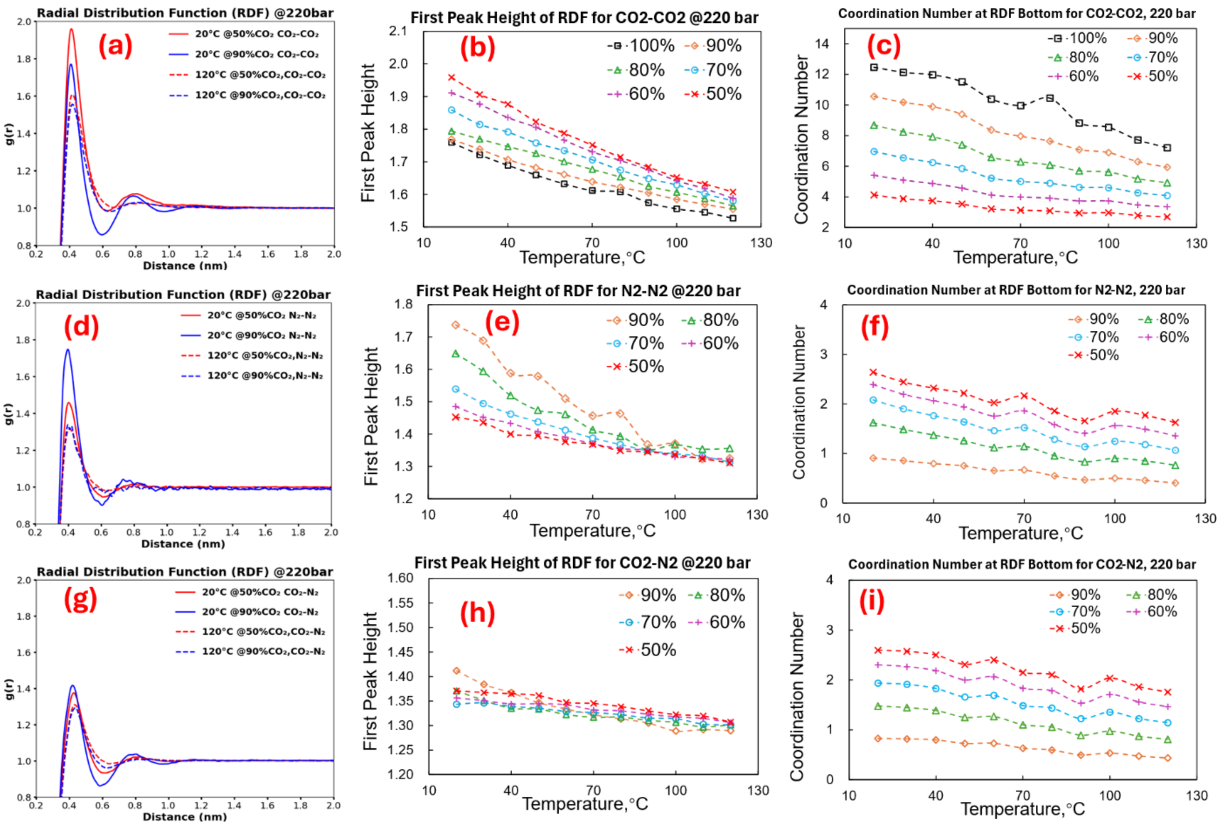

Supplementary Figure 1. Radial distribution function (RDF), RDF peak value, and coordination number for CO_2-CO_2, N_2-N_2, and CO_2-N_2.

Supplementary Note 2: Temperature and Pressure Gradient Profile

A comprehensive investigation of pressure and temperature was conducted to establish the T-P relation, as shown in Supplementary Table 1. Saline aquifers, conventional oil and gas reservoirs, shale, and coal beds are considered potential storage formations. In both sandstone and shale reservoirs, the common pressure gradient typically ranges between 90–120 bar km^{-1}, However, shale reservoirs in China exhibit higher pressure gradients, likely due to their greater burial depths. The temperature gradient range was not directly derived from the table data; instead, it was determined based on the global temperature distribution of sedimentary basins, making it a more

representative selection. The formulas for pressure gradient and temperature gradient are as follows:

$$T - T_0 = K_T \times D \quad (1)$$

$$P = K_P \times D \quad (2)$$

where T_0 is the initial temperature (°C), K_T is the temperature gradient (°C km^{-1}), K_P is the pressure gradient (bar km^{-1}), and D is the depth (km). P-T relation can be obtained by combining these two formulas.

Supplementary Table 1. Comparison of the Temperature and Pressure Feature for CO_2 Storage Formation

Project and/or Location	Type of Reservoir	Water Depth, m	Temperature gradient, °C/km	Pressure gradient, bar/km	Ref.
Sleipner, Norway	The Utsira, Sandstone	90	33	100~110	1,2
Decatur, USA	Mt. Simon, Sandstone	Onshore	18.2	~103	3,4
Nagaoka, Japan	The Haizume, Sandstone	Onshore	33	98	5,6
Tomakomae, Japan	Volcaniclastic rocks and sand	20~40	~33	92~120	7
In Salah, Algeria	Carboniferous Sandstone	Onshore	~32	~100	8
Louisiana, USA	The Haynesville Shale	Onshore	25~33	103~109	9
South China	The Longmaxi Shale	Onshore	~33	150	10
N/A	A typical sedimentary basin	N/A	30	150	11
Texas, USA	The Barnet Shale	Onshore	~33	~100	12
N/A	A natural Coal Seam	N/A	25	150	13

Supplementary References

1. Arts, R. *et al.* Monitoring of CO_2 injected at Sleipner using time-lapse seismic data. *Energy* **29**, 1383–1392 (2004).

2. Bickle, M., Chadwick, A., Huppert, H. E., Hallworth, M. & Lyle, S. Modelling carbon dioxide accumulation at Sleipner: Implications for underground carbon storage. *Earth and Planet. Sci. Lett.* **255**, 164–176 (2007).

3. Senel, O., Will, R. & Butsch, R. J. Integrated reservoir modeling at the Illinois Basin – Decatur Project. *Greenh. Gases* **4**, 662–684 (2014).

4. Finley, R. J. An overview of the Illinois Basin – Decatur Project. *Greenh. Gases* **4**, 571–579 (2014).

5. Sato, K. *et al.* Monitoring and simulation studies for assessing macro- and meso-scale migration of CO_2 sequestered in an onshore aquifer: Experiences from the Nagaoka pilot site, Japan. *Int. J. Greenhouse Gas Control* **5**, 125–137 (2011).

6. Mito, S., Xue, Z. & Ohsumi, T. Case study of geochemical reactions at the Nagaoka CO_2 injection site, Japan. *Int. J. Greenhouse Gas Control* **2**, 309–318 (2008).

7. Tanaka, Y. *et al.* Tomakomai CCS Demonstration Project of Japan, CO_2 Injection in Process. *Energy Procedia* **114**, 5836–5846 (2017).

8. Rutqvist, J., Vasco, D. W. & Myer, L. Coupled reservoir-geomechanical analysis of CO_2 injection and ground deformations at In Salah, Algeria. *Int. J. Greenhouse Gas Control* **4**, 225–230 (2010).

9. Nunn, J. A. Burial and Thermal History of The Haynesville Shale: Implication For Overpressure, Gas Generation, and Natural Hydrofracture. *Geol. Soc. (GCAGS) J.* v.1, 2012.

10. Pan, L., Xiao, X., Tian, H., Zhou, Q. & Cheng, P. Geological models of gas in place of the Longmaxi shale in Southeast Chongqing, South China. *Mar. Petrol. Geology* **73**, 433–444 (2016).

11. Zhang, H. & Cao, D. Molecular simulation of displacement of shale gas by carbon dioxide at different geological depths. *Chem. Eng. Sci.* **156**, 121–127 (2016).

12. Tang, X., Ripepi, N., Stadie, N. P., Yu, L. & Hall, M. R. A dual-site Langmuir equation for accurate estimation of high pressure deep shale gas resources. *Fuel* **185**, 10–17 (2016).

13. Li, X., Sun, X., Walters, C. C. & Zhang, T. H_2, CH_4 and CO_2 adsorption on Cameo coal: Insights into the role of cushion gas in hydrogen geological storage. *Int. J. Hydro. Energy* **50**, 879–892 (2024).
