# Supplementary material for: Economically viable geological CO2 storage from direct air capture has critical threshold of 70% CO2 concentration
Source: Commun Eng. 2025 Jul 15;4:127. doi: 10.1038/s44172-025-00468-5 (PMC12264126; doi:10.1038/s44172-025-00468-5)
Supplement: Supplementary file 3 — Description of Additional Supplementary Files [file 44172_2025_468_MOESM3_ESM.pdf]

Description of Additional Supplementary Files

File name: Supplementary Data 1

Description: Including initial and final configuration of molecular dynamics simulation at each concentration as well as the simulation settings.
